# Motivations, barriers and ethical understandings of healthcare student volunteers on a medical service trip: a mixed methods study

**DOI:** 10.1186/s12909-016-0618-0

**Published:** 2016-03-22

**Authors:** John Rovers, Kelsey Japs, Erica Truong, Yogesh Shah

**Affiliations:** College of Pharmacy & Health Sciences, Drake University, 2507 University Avenue, Des Moines, IA 50311 USA; Des Moines University, 3200 Grand Avenue, Des Moines, IA 50312 USA

**Keywords:** Volunteers, Students, Motivations, Barriers, Ethics, Medically underserved, Medical missions, Medical service trips

## Abstract

**Background:**

The motivation to volunteer on a medical service trip (MST) may involve more than a simple desire for philanthropy. Some volunteers may be motivated by an intrinsic interest in volunteering in which the context of the volunteer activity is less important. Others may volunteer because the context of their volunteering is more important than their intrinsic interest in volunteering. Furthermore, MSTs may pose a variety of ethical problems that volunteers should consider prior to engaging in a trip. This study evaluated the motivations and barriers for graduate health care students volunteering for an MST to either the Dominican Republic or Mississippi. Volunteers’ understanding of some of the ethical issues associated with MSTs was also assessed.

**Methods:**

Thirty-five graduate health professions students who volunteered on an MST were asked to complete an online survey. Students’ motivations and barriers for volunteering were assessed using a 5-point Likert scale and Fisher’s exact test. Ethical understanding of issues in volunteering was assessed using thematic analysis.

**Results:**

Students’ motivations for volunteering appeared to be related to the medical context of their service more than an inherent desire for volunteer work. Significant differences were seen in motivations and barriers for some student groups, especially those whose volunteer work had less opportunity for clinical service. Thematic analysis revealed two major themes and suggested that students had an empirical understanding that volunteer work could have both positive and negative effects.

**Conclusions:**

An understanding of students’ motivations for volunteering on an MST may allow faculty to design trips with activities that effectively address student motivations. Although students had a basic understanding of some of the ethical issues involved, they had not considered the impact of a service group on the in-country partners they work with.

## Background

A simple definition for volunteering would be, “any activity in which time is given freely to benefit another person, group or organization” [1]. Service on a medical mission trip is an increasingly common way to volunteer one’s services. Volunteers on these trips are frequently members of religious, civic or professional groups who have an interest in health in medically underserved areas. Groups raise funds, recruit volunteers, purchase medical supplies and provide medical and/or public health services. In most cases, groups return to the same area annually to provide short-term services [2].

Although the term “medical mission trip” is in common usage, it suggests a religious or military impetus for the volunteer activity. Absent such an impetus, the term “medical service trip” (MST) may be preferable and will be used throughout this paper.

Health care professionals from the developed world are frequent volunteers on MSTs to areas with medically underserved populations. Such volunteers often include physicians, nurses, pharmacists and other providers as well as students from these respective disciplines [3, 4]. Estimates of the efforts expended on MSTs suggest that the United States alone has over 500 organizations that send up to 6000 trips per year [5, 6].

MSTs are often organized and operated by universities whose students then provide medical care, under supervision, to medically underserved, often fragile populations. Student-provided medical care may carry accompanying risks to both patients and students, which suggests there are inherent ethical issues for MSTs. Consequently, universities should have an understanding of why their students wish to participate on an MST, their understanding of the ethics of participation, as well as the risks involved. Given the size of this volunteer effort, and the attendant risks and ethical concerns, studies on volunteering for an MST should be of some interest.

Existing studies of volunteer experiences suggest several conceptual problems that may be of interest. First, when asked why they volunteer, most people simply say they want to help others. According to Shye, this explanation poses an interesting problem for research into volunteering since helping others is essentially the definition of volunteering [7]. Consequently, studies of volunteers’ motivations can become somewhat circular exercises.

Second, although popular usage of the term volunteering suggests that volunteers’ motivations are primarily altruistic, the literature suggests that volunteers’ motivations may not be as simple as that. Wilson notes that two perspectives on volunteering predominate [1]. The first perspective posits that a volunteer’s reasons for volunteering may be complex, while the context of the volunteer work is simply background. This kind of volunteer has an inherent desire to volunteer while the specific volunteering activity matters less. Here, the inherent desire to volunteer matters most.

The second perspective treats the volunteer as having fairly simple motives for volunteering, but the context in which the volunteering occurs is complex. For this kind of volunteer, the specific activity to which volunteering efforts will be devoted drives the interest in volunteering more than the inherent belief that volunteering is important. Here, the context for volunteering matters most.

For example, the first perspective takes a subjectivist approach in which a person may believe that volunteering is a socially responsible activity that all citizens should engage in while also believing that where one chooses to volunteer (e.g. political party, church group) matters less. This approach is primarily interested in the motives for volunteering.

The second perspective takes more of a behaviorist approach. It assumes the volunteer is a rational actor who makes decisions to volunteer depending on the costs and benefits of their service within the context of their individual and social resources. For example, a student may volunteer for a project to help the homeless because they have the resources to do so and also have the hope that the service will be helpful for their application to graduate school. At the same time, they may choose not to volunteer for a socially controversial group they would otherwise support if they were concerned it could come at the cost of damaging the graduate school application. This approach is primarily interested in the context for volunteering.

Others have applied the behaviorist perspective to express ethical concerns about how the context of volunteer work affects motivation to do international health and development work, which would include MSTs. Diprose states that short-term volunteer projects have shifted the emphasis from knowledge transfer to local communities to the personal growth of the volunteer [8]. Smith and Laurie state that, “…a focus on professional skills needs in the Global South is superseded by a growing emphasis on the needs of the individual volunteer and their own personal development” [9]. They also express concerns at what they describe as a tacit acknowledgement by agencies offering volunteering opportunities that sending young, partly trained volunteers may not offer significant benefit in the Global South.

Some facets of development theory also offer an opportunity to evaluate the motivations and ethics behind international volunteer work. One aspect of development theory is modernization theory, which holds that social scientists can identify the health, social and other factors that allowed Western countries to develop into modern economies. Development agencies and non-governmental organizations (NGOs) may then send volunteers abroad to introduce the missing factors (e.g. health care services, including MSTs) into developing world systems to aid their development [10]. Critics of modernization theory often cite dependency theory and claim that this type of health and development work is ethnocentric, primarily reflects the values and priorities of Western agencies and volunteers, and reinforces the deleterious effects of a colonial past.

The ethical concerns expressed in the literature should be of interest to universities offering MSTs to their students. Universities should ensure that student motivations to enhance their practice skills do not outweigh any benefits that accrue to the community served. Students must be adequately supervised by licensed practitioners. Finally, there should be an opportunity for students to increase their ethical understanding of how MSTs may actually harm communities.

Although the literature is replete with studies of volunteers in various situations, comparatively few are investigations of students in the health professions volunteering on MSTs in an international setting. Bimstein et al. surveyed dental students who volunteered to provide care in a number of developing countries [11]. Brown et al. evaluated the experiences of student pharmacists on both introductory and advanced clinical placements [4]. They found that some student beliefs and attitudes changed, but they did not evaluate any motivations or barriers that students may have had. Neither study evaluated students’ understanding of the ethics of serving on MSTs.

## Objectives

The conceptual and ethical issues surrounding volunteer work, coupled with the paucity of research into health professions students’ volunteer work, suggest this may be a fruitful area for research. The results of such studies should prove useful for universities to better prepare students for volunteering on MSTs and enhance their understanding of some of the ethical concepts inherent in such work.

The primary objective of this study is to evaluate the motivations and barriers of healthcare students who have volunteered on an MST to an underserved community. Do such students volunteer out of an inherent desire to volunteer, or is the medical context of the volunteering activity more important? The secondary objective is to determine students’ understanding of some of the ethical considerations surrounding MSTs.

## Methods

### Subject selection

Study subjects were first or second year students in osteopathic medicine, podiatric medicine, or physician assistant studies at a graduate health sciences university in a Midwestern US state. These students volunteered for an MST to either the Dominican Republic or rural Mississippi during March Break 2015.

All subjects were over the age of 18, and speak, read and write English. Prior to serving on the MST, student potential volunteers underwent a selection process consisting of an interview, an application essay, evaluation of their foreign language skills and an evaluation of any extra or co-curricular activities. Approximately 50 students applied to participate on an MST, of whom 35 were selected and who received an email after completion of the trip requesting them to complete an online survey.

### Description of MSTs

The Dominican Republic trip was a one week experience to provide medical care in Monte Cristi, a city on the northwest coast, near the Haitian border. Patients were low income workers on banana plantations and their families. Dominican patients spoke Spanish while Haitian patients spoke mainly Creole, so local interpreters were provided for those students who spoke only English. The sponsoring university developed the MST in partnership with a local non-governmental organization (NGO) that provided most of the health care services in the area. Under the supervision of licensed, American health care professionals, students provided a variety of health care services including physical exams, diagnosis of illness, prescribing medication, patient education, dispensing medication, and basic laboratory services. Student costs were approximately $750 (USD) for airfare to the Dominican Republic plus $900 (USD) for the local NGO to cover accommodation, meals and local transportation. Some of these costs (typically airfare) were paid by the sponsoring university and not all students paid these full amounts out of pocket.

The Mississippi trip was a week long experience designed to provide limited health services and establish connections for future MSTs in Mississippi, a largely poor, rural state in the southern USA. Mississippi patients were ethnically diverse with both white and African American patients, who frequently live at or below the federal poverty level and who have limited access to health care services. Students’ opportunities to provide clinical patient care were designed to be more limited than the Dominican Republic trip. Students assisted in health screenings, and quality improvement projects at the health clinic. Consequently, more of their experience was spent in observation of locally provided healthcare. Students paid approximately $250 (USD) for accommodations and meals. Transportation costs were donated by an outside party.

### Data collection

We used a mixed methods approach to collect both quantitative and qualitative data. Quantitative methods allow for a convenient, reproducible way to measure subjects’ motivations and barriers, while qualitative methods permit investigators to explore subjects’ perceptions and lived experiences of their volunteering.

After returning from their MSTs, students were asked to complete an online survey using an instrument posted on the Qualtrics ® web site. Questions evaluating subjects’ motivations and barriers for volunteering on the MST were modified from the questions used in Bimstein’s study of dental students [11]. According to Shye, use of pre-determined questions minimizes the potential for social desirability bias in subjects’ responses about motivations and barriers [7]. The study was approved by the Drake University Institutional Review Board and all subjects gave informed consent prior to completing the survey.

Reflection questions evaluating subjects’ understanding of potential ethical issues for MSTs were asked in a free text format. Subjects were asked about any previous coursework or self-study they had completed on development theory as well as their understanding of development theory. Subjects also completed a demographic section that asked about age, sex, course and year of study, and previous experience as a volunteer on MSTs.

The survey instrument is included in the Appendix.

### Data analysis

Quantitative data were imported into Stata v9.2 for analysis. Demographic results were tabulated, as was the median response for the 22 questions regarding motivations and barriers. Fisher’s exact test was used to determine if any of the demographic differences within groups (e.g. male/female—the independent variables) resulted in differences in subjects’ motivations and barriers (the dependent variables). p values of ≤ 0.05 were considered significant.

The responses to reflection questions were imported into Microsoft Word for qualitative analysis by a team of three investigators (JR, KJ, ET). Transcripts were analyzed using thematic analysis [12]. Each investigator read the transcripts of the responses to each of the five reflection questions independently and developed a preliminary codebook used to assign descriptive codes to segments of the text. Codes were derived from the data using a positivist approach. Team members met weekly to reach consensus on the definition of and use for each specific code and a final codebook was created, which was used to re-code each transcript. The re-coded transcripts were then read to identify overarching themes represented in the data. As before, team members identified themes individually, and then reached consensus on the final themes identified.

## Results

Thirty-five students participated on an MST. Eleven students went to Mississippi and 24 to the Dominican Republic. Thirty-six individuals opened the survey link, 35 of whom were students and one was a medical provider sent the survey link in error and whose responses are not included in the results. This yielded 33 usable responses (94.2 %). The age of subjects was 25.35 +/− 2.65 years (mean +/− SD). Most subjects were female, studying osteopathic medicine and carried out their MST in the Dominican Republic. Fifteen of the 33 (45 %) had completed a previous MST. Only about one third of subjects had undertaken any study or had familiarity with development theory. A demographic description of the subjects is shown in Table 1.

**Table 1:** Subject Demographics

Independent Variables	Number (*N* = 33)	Percent
Sex		
Male	11	32
Female	22	68
Course of Study		
DO	22	67
DPM	10	30
PA	1	3
Year of Study		
First Year	17	52
Second Year	16	48
Participation on MST		
Yes	15	47
No	18	53
Number of Trips Previously		
Participated On		
1	6	18
2	7	21
3	1	3
> 3	1	3
Trip Destination		
Dominican Republic	22	68
Mississippi	11	32
Previous Study of Development		
Theory		
Yes	10	30
No	23	70
Familiar With Modernization Theory		
Yes	11	33
No	22	67
Familiar With Dependency Theory		
Yes	13	39
No	20	61

### Quantitative results

The median response (from 1 to 5) for each motivator or barrier is shown in Table 2.

**Table 2:** Barriers and Motivations

Dependent Variables - Motivations/Barriers	Median Response^a^
Motivations	
Interacting with other cultures	5
Interacting with other health professionals	5
Educational opportunity	5
Philanthropy/helping others	5
Develop my clinical skills	5
Improved personal confidence	4
Pure enjoyment	4
Improved interpersonal skills	4
Opportunity for travel	4
Improved foreign language skills	4
Help build my résumé/CV	3
Receiving class credit	2
Someone asked me to volunteer	1
Barriers	
Cost of the trip	4
Time commitment	4
Substandard working conditions	3
Substandard living conditions	3
Language barriers	3
Paperwork/administrative barriers	3
Exposure to infectious diseases	3
Threat of crime	2
Prefer to use free time for leisure, not volunteering	2

^a^Response scale of 1 to 5, where 1 = not important at all, 5 = very important

Given the differences within the independent variables of each demographic group shown in Table 1, Fisher’s exact test was used to determine if there were differences in responses to the questions on the dependent variables for motivations and barriers. The results are shown in Tables 3, 4, 5, 6, and 7.

**Table 3:** Significant difference in motivations/barriers by sex

Desire for philanthropy							
	1^a^	2^a^	3^a^	4^a^	5^a^	Total	*p* value
Male	0	0	0	4	7	11	0.033
Female	0	0	0	1	21	22	
Someone asked me to go							
	1^a^	2^a^	3^a^	4^a^	5^a^	Total	*p* value
Male	7	4	0	0	0	11	0.048
Female	15	1	5	1	0	22	

^a^Number of respondents answering 1 (not important at all) 2 Not very important 3 (Neither important nor unimportant) 4 (Fairly important) 5 (Very important)

**Table 4:** Significant difference in motivations/barriers by course of study

Improved personal confidence							
	1^a^	2^a^	3^a^	4^a^	5^a^	Total	*p* value
DO^b^	0	3	9	5	5	22	0.019
DPM^c^	1	0	0	7	2	10	
PA^d^	0	0	0	1	0	1	

^a^Number of respondents answering 1 (not important at all) 2 Not very important 3 (Neither important nor unimportant) 4 (Fairly important) 5 (Very important)

^b^Doctor of Osteopathic Medicine

^c^Doctor of Podiatric Medicine

^d^Physician Assistant

**Table 5:** Significant difference in motivations/barriers by year of study

Help build my CV							
	1^a^	2^a^	3^a^	4^a^	5^a^	Total	*p* value
1st year	0	2	3	9	3	17	0.002
2nd year	2	0	11	2	1	16	
Develop my clinical skills							
	1^a^	2^a^	3^a^	4^a^	5^a^	Total	*p* value
1st year	0	0	0	4	13	17	0.049
2nd year	0	1	0	9	6	16	

^a^Number of respondents answering 1 (not important at all) 2 Not very important 3 (Neither important nor unimportant) 4 (Fairly important) 5 (Very important)

**Table 6:** Significant difference in motivations/barriers by participation on previous MST

Desire to receive academic credit							
	1^a^	2^a^	3^a^	4^a^	5^a^	Total	*p* value
Previous participation	8	6	1	0	0	15	0.018
No previous participation	6	2	8	2	0	18	
Improved personal confidence							
	1^a^	2^a^	3^a^	4^a^	5^a^	Total	*p* value
Previous participation	0	0	5	4	6	15	0.037
No previous participation	1	3	4	9	1	18	
Improved interpersonal skills							
	1^a^	2^a^	3^a^	4^a^	5^a^	Total	*p* value
Previous participation	0	0	2	5	8	15	0.037
No previous participation	0	1	0	13	4	18	

^a^Number of respondents answering 1 (not important at all) 2 Not very important 3 (Neither important nor unimportant) 4 (Fairly important) 5 (Very important)

**Table 7:** Significant difference in motivations/barriers by MST service destination

Interact with other health professionals							
	1^a^	2^a^	3^a^	4^a^	5^a^	Total	*p* value
Dominican Republic	0	0	0	5	17	22	0.028
Mississippi	0	0	0	7	4	11	
Educational Opportunity							
	1^a^	2^a^	3^a^	4^a^	5^a^	Total	*p* value
Dominican Republic	0	0	0	2	20	22	0.002
Mississippi	0	0	0	7	4	11	
Desire for philanthropy							
	1^a^	2^a^	3^a^	4^a^	5^a^	Total	*p* value
Dominican Republic	0	0	0	0	22	22	0.002
Mississippi	0	0	0	5	6	11	
Develop my clinical skills							
	1^a^	2^a^	3^a^	4^a^	5^a^	Total	*p* value
Dominican Republic	0	1	0	4	17	22	0.002
Mississippi	0	0	0	9	2	11	
Pure enjoyment							
	1^a^	2^a^	3^a^	4^a^	5^a^	Total	*p* value
Dominican Republic	1	0	1	9	11	22	0.001
Mississippi	0	5	6	0	0	11	
Improved interpersonal skills							
	1^a^	2^a^	3^a^	4^a^	5^a^	Total	*p* value
Dominican Republic	0	1	1	9	11	22	0.041
Mississippi	0	0	1	9	1	11	
Improved language skills							
	1^a^	2^a^	3^a^	4^a^	5^a^	Total	*p* value
Dominican Republic	0	1	5	5	11	22	0.001
Mississippi	4	0	5	2	0	11	
Time commitment							
	1^a^	2^a^	3^a^	4^a^	5^a^	Total	*p* value
Dominican Republic	1	9	1	10	1	22	0.013
Mississippi	0	1	4	3	3	11	
Fear of crime							
	1^a^	2^a^	3^a^	4^a^	5^a^	Total	*p* value
Dominican Republic	4	8	1	7	2	22	0.038
Mississippi	3	2	5	1	0	11	

^a^Number of respondents answering 1 (not important at all) 2 Not very important 3 (Neither important nor unimportant) 4 (Fairly important) 5 (Very important)

There were significant differences in motivations and barriers for several demographic groups. Women were more likely to be motivated by a desire for philanthropy (*p* = 0.033) or because someone asked them to go (*p* = 0.048) than men. (Table 3) Podiatric medicine students were more likely to be motivated by an opportunity for improved personal confidence compared to osteopathic medicine or physician assistant students (*p* = 0.019). (Table 4) Students in their first year were more motivated by a desire to build their CV (*p* = 0.002) and improve their clinical skills (*p* = 0.049) than second year students. (Table 5) Students who had not participated in a previous MST were more motivated by receiving academic credit (*p* = 0.018), improving their personal confidence (*p* = 0.037) and improving their interpersonal skills (*p* = 0.037) than those with previous MST experience. (Table 6) Compared to those who went to the Dominican Republic, students on the Mississippi trip were less likely to be motivated by a desire to work with other health professionals (*p* = 0.028), educational opportunity (*p* = 0.002), a desire for philanthropy (*p* = 0.002), developing their clinical skills (*p* = 0.002), pure enjoyment (*p* = 0.001), improved interpersonal skills (*p* = 0.041) and improved language skills (*p* = 0.001). (Table 7) Students on the Mississippi trip were also less likely to rate time commitment (*p* = 0.013) and fear of crime (*p* = 0.038) as barriers. Age and the number of previous MSTs did not have any significant effect on motivators or barriers.

### Qualitative results

**Fig. 1:**
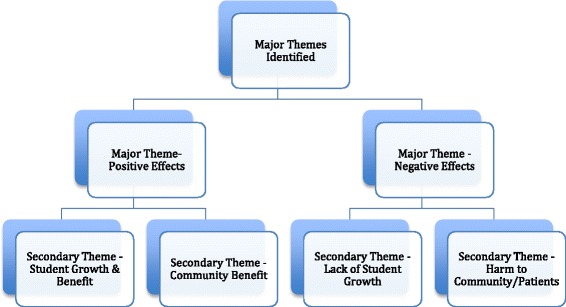
Qualitative Themes and Secondary Themes Identified

Thematic analysis of the free text responses revealed two major themes and several secondary themes. The first major theme was that MSTs can have positive effects while the second major theme was that MSTs can also have negative effects. Each major theme also contained two secondary themes. These secondary themes revealed to whom or to what the positive and negative effects accrued. A diagram of how themes were identified and are related to each other is shown in Fig. 1.

Secondary themes were identified as positive effects that accrued to both students and the community. Students understood themselves to be major beneficiaries of the MST in that they experienced personal growth, a better understanding of poverty, and an understanding of the limits of their clinical competence. Effects on the community were improved access to medical care and a sense of hope that outsiders were interested in their wellbeing.

Secondary themes of negative effects showed they accrued mostly to the community. Students appreciated the potential for clinical harm, the power differentials between themselves and their patients, and the potential for MSTs to create dependencies in the community. Negative effects for students were more subtle and included a lack of personal growth and a continued understanding of poverty as only a material want.

#### Major theme of positive effects

Students saw themselves as being the primary beneficiary of the MSTs. They recognized that they developed broader perspectives about the world, themselves, the practice of medicine and the nature of poverty during the trip.

#### Secondary theme of positive effects on students

In some cases, student growth reflected better understanding of health care in an underserved area:

I learned much about the cultural, historical, and sociopolitical context of the Dominican Republic, especially the area we were in, which was near the border of Haiti. Understanding the cultural context helped me understand why some groups receive health care and some are incredibly under-served. I went on this trip expecting to have my perceptions of Monte Cristi and the DR expanded, challenged, and changed. I would say the trip met my expectations in all three ways.

(28-year-old female osteopathic medicine student)

In other cases, student growth occurred at a deeper, more personal level in which the student drew comparisons between the communities served in and the student’s own life in the United States:

I think the thing that strikes me the most when coming to these communities however, is that I know what it is like to live in excess, to have the luxury of having clean, sanitary, running water, to get food whenever convenient, to have a safe home to live in, and I know these Dominican communities are aware they live in poverty, but I don’t believe they know how different their life really is from ours. The conditions they live in are normal for them, and although they are substandard for us outsiders, their perspective is much different than ours.

(25-year-old female osteopathic medicine student)

A third area of student growth was in recognizing that poverty is more than just a lack of wealth and material goods:

I did notice that the infrastructure is poorly built in the area. What surprised me the most is how much segregation still exists in today’s society. Crossing the bridge could mean entering a community where the condition is no better than a third world country.

(23-year-old male podiatric medicine student)

Poverty is often systemic and out of the scope of actually being fixed by short term trips- we can only cover some of the issues with a bandaid.

(23-year-old female podiatric medicine student)

Since all students on both trips had limited clinical experience prior to the MST, students also benefited by being able to see what clinical skills they already possessed:

As a first year student I was pleasantly surprised that I was equipped to evaluate many of the cold like symptoms that presented. As well as perform basic physical exam skills that I had learned in my clinical medicine course.

(24-year-old female podiatric medicine student)

Students also recognized the limits of their clinical competence. In some cases, this was reflected as being dependent upon the licensed providers on the MST:

I have also not had any pharmacology courses yet, so working in pharmacy was very challenging, however that was one area I learned the most in and am very grateful for being able to experience that.

(25-year-old female osteopathic medicine student)

In other cases, students recognized the limits of their practice skills since they had limited opportunity to treat patients. All such students participated on the Mississippi trip:

As a first year, I felt like I was not able to do a whole lot of clinical practice as there were restrictions.

(25-year-old male podiatric medicine student)

#### Secondary theme of positive effects on communities

The communities that hosted students were also found to gain some positive effects. There was a general belief that the MST contributed towards building a better community:

I believe a potential benefit was continuing to establish a more concrete health care system to these small communities, whom, without us would not have access to this level of medical care. I believe the community in general received these benefits, especially those who needed medical referrals.

(25-year-old female osteopathic medicine student)

In most cases, however, students had a more nuanced impression of community effects and often balanced effects on the community with effects on the students themselves. Sometimes, the effects on students and communities were judged to be about equal:

The benefits of my volunteering consisted of helping a greater effort, one that was outside of myself that focused on the needs to the population we served. Naturally, the patient population benefited from health care services, but I also benefited from this experience in terms of my education and broadening my experiences/knowledge in terms of global health care.

(24-year-old female podiatric medicine student)

At other times, students believed that effects on the community outweighed effects on the students, while in some instances, the students felt the positive effect accrued more towards themselves than the community:

I believe that simply our presence and our good intentions gave the local people hope. The locals appreciated that we gave up our spring break to volunteer and educate them. I think that they really appreciated that.

(24-year-old female osteopathic medicine student)

I believe the people we saw did indeed benefit from our trip, but perhaps more on a short term scale. Its [*sic*] the people on the trip who received more long lasting benefits of learning a new culture, clinical skills, and having these patients leave an ever lasting impact in our minds and our spirits.

(26-year-old female osteopathic medicine student)

#### Major theme of negative effects

Most but not all students appreciated that MSTs may have negative effects and that such negative effects are more likely to accrue to the community than themselves.

#### Secondary theme of negative effects on communities

Given that these were MSTs, clinical harm to patients was cited as a potential negative effect:

We don’t know if people ever really took the medications as directed, so there’s always a potential for medication errors. We also don’t know the rates of endemic diseases like hepatitis, and so even meds as benign as acetaminophen can be problematic.

(28-year-old female osteopathic medicine student)

As with potential community positive effects, there were also several more nuanced observations about harm causing effects. Students recognized that language barriers, power differentials and cultural barriers also had the potential to result in harm to patients:

I think our lack of cultural competency was the biggest potential harm to the patients—in a number of ways. We didn’t really ever have confirmation that our patients understood what we were saying, unless we could speak directly to them in their language, and most of us couldn’t. We have different cultural conceptions of disease, so it makes history-taking challenging at best.

(28-year-old female osteopathic medicine student)

We were a group of educated foreigners providing medications and medical education to vulnerable populations which sets us up for the potential of causing many harms to the communities we served. There is a risk of inadequate counseling due to language barriers alone; compound that with the limited time we spent with each patient, the cultural differences, and ‘power’ differences, it seems almost impossible to adequately counsel patients about medication side effects, adequately obtain a thorough health history, and adequately education [*sic*] patients about their medical condition. The use of Dominican translators did reduce the potential of harm, but because these communities regularly go weeks without a doctors [*sic*] presence, potentially harmful medication side effects may go unrecognized, and potentially incorrect adherence to medications may continue without recognition.

(26-year-old female osteopathic medicine student)

Beyond any patient related harm, harm to the host community was also a potential negative effect identified by students. The economic impact of a large group of foreigners bringing in substantial quantities of medical and other supplies had the potential to damage local businesses or medical providers:

I think one of the potential harms of our volunteer work was taking business away from local businesses. By having us bring hygiene packs, sunscreen, lotion, and other daily essentials, it takes away business from local stores who sell those products.

(26-year-old female osteopathic medical student)

Students also expressed concern that MSTs could create dependencies in local communities. Such dependences could develop either at the level of the local clinic, or at the level of government policies on health care spending:

Sometimes the nurses at the free clinic would rely too much on the medical students for providing certain services. While this is a good experience, it may jeopardize the patients’ wellbeing.

(23-year-old male podiatric medicine student)

One could argue that because a group like [*redacted*] provides care, the government is less incentivized to have a functional health system accessible to all. I disagree with this, but still think it’s a potential harm.

(28-year-old female osteopathic medicine student)

#### Secondary theme of negative effects on students

Potential negative effects that accrued to students were more subtle, but nevertheless existed. Not all students had the experience of personal growth as a result of working in a medically underserved setting:

I was pretty indifferent to the poverty. It was of a caliber that I had experienced before, just in a different region.

(24-year-old male osteopathic medicine student)

Not all students came to see poverty as multi-dimensional and some continued to conceptualize it in the context of material want:

For example, most Dominican families had cell phones and a few places had televisions, which is definitely not what I expected before coming to these communities. After several discussions however, I began to realize even though these people are living in extreme poverty, they are willing to spend a little extra money on commodities that improve their quality of life, just as us Americans do here in the United States (albeit us being on a much more gluttonous level).

(25-year-old female osteopathic medicine student)

## Discussion

Wilson states that the motivation to volunteer can either be driven by the volunteer’s intrinsic motives or by the context of the volunteering experience [1]. Our quantitative results suggest that health care students’ participation on an MST appears to be context dependent and that the medical nature of the volunteering opportunity and activities performed may be more important than the simple desire to volunteer.

### Motivations

The highest rated (5/5) motivators to participate on an MST were the opportunity to interact with other cultures, to work with other health professionals, the educational opportunities, the chance to improve clinical skills, and the desire for philanthropy. (Table 2) Volunteer activities that allow volunteers to work with other cultures, gain educational benefits and fulfill a desire for philanthropy can be performed nearly anywhere. But for health care students, improving clinical skills and the opportunity to work with other health care professionals are motivations that are best fulfilled on an MST. Without a control group to compare the motivations of students undertaking volunteer work other than an MST to volunteers on an MST, these results are not definitive. However, since several of the students’ highest rated motivations were ones that could only be fulfilled on an MST, the results are at least suggestive that the desire to volunteer was context dependent.

Similarly, the three lowest rated motivators were the opportunity to build one’s CV (3/5), the desire for academic credit (2/5) and because someone asked (1/5). These motivators are ones that could also have been readily satisfied by volunteering in a non-medical setting. Yet, these volunteers gave up a Spring Break vacation and spent between $250 and $1650 (USD) to volunteer for an MST. There appears to be something in the context of an MST that motivated students to undertake an expensive, time consuming activity that may be helpful in their professional training.

### Barriers

The highest rated (4/5) barriers to volunteering on an MST were the cost and time involved. Both trips involved time and costs related to travel to either the Dominican Republic or Mississippi. These barriers could have readily been overcome by students participating in volunteer activities in their home communities. As noted above, they nevertheless participated in time consuming and expensive volunteer work despite the barriers. The context of the volunteer work appears to overcome the barriers.

Fifteen of 33 (45 %) of the respondents had volunteered on previous MSTs. Other authors have stated that up to 30 % of medical students engage in some kind of MST experience [5]. Although we have no comparative data to demonstrate their interest in non-MST volunteer work, it appears that health care students have a substantial interest in MSTs. We would surmise that the medical nature of the trip is students’ primary motivation. These results are not definitive, but they appear to support Wilson’s notion that volunteering may be context dependent [1].

Our quantitative results also show that there were significant differences in how some students rated their barriers and motivations relative to their student colleagues. (Table 3) For example, students in their first professional year of training were more likely to be motivated by a desire to build their CV and to improve their clinical skills. This is likely a result of first year students having less experience than more senior students and being anxious to develop their patient care skills. In addition, applications to volunteer on the MST are due only a few weeks after first year students have started medical school, which may add to their desire to begin their professional training as soon as possible.

The most common differences between groups were seen when comparing students on the Dominican Republic trip with those on the trip to Mississippi. These trips were designed differently so that students on the Mississippi trip had fewer opportunities to provide hands-on patient care and to work closely with licensed health care providers. As a result, these students were less motivated by clinical, education, and career development opportunities than students on the Dominican Republic trip.

An awareness of these kinds of differences may be helpful to faculty members or others who design and develop MSTs for students. Knowing that first professional year students may be more motivated by the chance to develop their practice skills can be helpful when selecting students for a trip or making work assignments once a group is in-country. Similarly, if a trip offers mostly shadowing and observational experiences, it behooves faculty to determine if student motivations to volunteer are congruent with the actual activities they will participate in.

Our qualitative results suggest that student volunteers had a reasonably good grasp of some of the ethical aspects of serving on an MST. They understood their volunteer activities could result in both positive and negative effects for themselves, their patients, and the community. Students were sensitive to the fact that often, they were the primary beneficiaries of their own volunteer service. They grew personally and developed professionally as a result of their volunteering.

Students’ awareness of how their volunteer work served students themselves supports the concerns of Diprose and Smith and Laurie that short-term medical volunteer work too often has the ethical limitation of being designed to meet the needs of the volunteers, rather than the communities served [8, 9]. And as Greig et al. suggest, simply importing first world health care into underdeveloped health systems may not fix many problems [10]. Student responses to the reflection questions showed that they recognized these ethical concerns.

Despite the fact that only about a third of them had undertaken any study in health and development, students did appear to have an empirical understanding of the concerns expressed by critics of volunteer work. Beyond seeing only the risk for harm due to medical mis-adventuring, there was an understanding that volunteer work could create dependencies and distortions in the local economy and that underlying problems, often stemming from poverty, may not be addressed. As one student put it, “…we can only cover some of the issues with a bandaid.”

There is nothing in our results that would directly address the concerns of critics of volunteer MSTs, but the evidence suggests that student volunteers do have an understanding of the limitations of such trips.

There was one ethical concern noted in the literature that students did not address. As for the Dominican Republic trip, MST volunteers frequently must partner with local NGO’s who remain and work in-country after the volunteers have left. Berry has expressed concerns that the objectives of such NGO’s and MST volunteers may often be at odds with each other, which is especially problematic for NGO’s that depend on the fees they charge visiting groups for their financial sustainability [13]. Solheim and Edwards point out that volunteers will require local assistance with transportation, lodging, meals and other ground support [14]. Without careful planning and attention, MST volunteers can quickly overwhelm their local host’s ability to provide needed support services. None of the students replying to this survey appeared to have considered some of the ethical concerns related to the relationship volunteers need to have with their local hosts. These are ethical concerns that faculty who plan MSTs should be careful to address in pre-departure briefings.

## Limitations

We note there are a number of limitations to this study. The sample size was small and although study subjects were drawn from one school, the type of volunteer work differed according to if they traveled to Mississippi or the Dominican Republic. Study subjects were surveyed after their return from their MST. A pre-post design may have provided different results. It is possible that students’ motivations, barriers, and ethical understandings were not affected by their participation in the MST. Thirty-three total responses for the study may have resulted in some demographic groups being too small for the study to be adequately powered to measure small differences. Although a control group would have been ideal, the ability to identify and study control students who did not volunteer for an MST was beyond our technical limitations. Finally, our qualitative results may not be generalizable to other schools, practice settings or volunteer opportunities, which is consistent with the nature of qualitative data [15].

## Conclusions

The motivations and barriers for health professional students to volunteer on an MST may depend on the context of the trip and the opportunities it provides, rather than an intrinsic desire to volunteer. Demographic differences between volunteers may be helpful in designing MSTs. Student volunteers generally had an empiric understanding of the ethical consequences of their volunteer work, but were unaware of how volunteer work may impact local partners of volunteer groups.

### Data availability

The dataset supporting the conclusions of this article is available in the figshare repository with the unique persistent identifier and hyperlink to the dataset of https://figshare.com/articles/Volunteering_Data_pdf/3113356

## Appendix

### Survey questions

On a scale of 1 to 5 where 1 is not important at all and 5 is very important, indicate how much each of the items below influenced your decision to volunteer on this medical service trip:

1. Cost of the trip
2. Receiving class credit
3. Interacting with other cultures
4. Time commitment
5. Improved personal confidence
6. Threat of crime
7. Interacting with other health professionals
8. Substandard working conditions
9. Substandard living conditions
10. Educational opportunity
11. Philanthropy (helping others)
12. Language barriers
13. Help build my résumé
14. Paperwork/administrative barriers
15. Develop my clinical skills
16. Pure enjoyment
17. Improved interpersonal skills
18. Opportunity for travel
19. Improved foreign language skills
20. Exposure to infectious diseases
21. Prefer to use free time for leisure, not volunteering
22. Someone asked me to volunteer

In the space below, please answer the following reflection questions:

1. In what ways, if any, did your volunteer experience expand, challenge or change your perceptions of the community you volunteered in?
2. What was your reaction to seeing people living in poverty?
3. How adequate were your clinical practice skills to provide volunteer health care?
4. What do you believe the potential benefits of your volunteer work on this service trip were and who received such benefits?
5. What do you believe the potential harms of your volunteer work on this service trip were and who was at risk for such harms?

Please answer the following questions by checking the appropriate box:

1. Have you ever studied development theory either formally in a class or informally on your own?
2. Are you familiar with the concept of Modernization Theory?
3. Are you familiar with the concept of Dependency Theory?

## Abbreviations

MST: medical service trip
NGO: non-governmental organization

## References

[1] Wilson J (2000). Volunteering. Ann Rev Sociol.

[2] Rovers J, Andreski M, Gitua J, Bagayoko A, DeVore J (2014). Expanding the scope of medical mission volunteer groups to include a research component. Global Health.

[3] Laleman G, Kegels G, Marchal B, Van der Roost D, Bogaert I, Van Damme W (2007). The contribution of international health volunteers to the health workforce in sub-Saharan Africa. Hum Res Health.

[4] Brown DA, Fairclough JL, Ferrill MH (2012). Planning a pharmacy led medical mission trip, part 4: an exploratory study of student experiences. Ann Pharmacother.

[5] Snyder J, Dharams S, Crooks VA (2011). Fly by medical care: conceptualizing the global and social responsibilities of medical tourists and physician voluntourists. Global Health.

[6] Maki J, Qualls M, White B, Kleefield S, Crone R (2008). Health impact assessment and short-term medical missions: a methods study to evaluate the quality of care. BMC Health Serv Res.

[7] Shye S (2010). The motivation to volunteer: a systematic quality of life theory. Soc Indic Res.

[8] Diprose K (2012). Critical distance: doing development education through international volunteering. Area.

[9] Smith MB, Laurie N (2011). International volunteering and development: global citizenship and neoliberal professionalization today. Trans Inst Br Geogr.

[10] Greig A, Hulme D, Turner M (2007). Challenging Global Inequality: Development Theory and Practice in the 21st Century.

[11] Bimstein E, Gardner QW, Riley JL, Gibson RW (2008). Educational, personal and cultural attributes of dental students’ humanitarian trips to Latin America. J Dent Educ.

[12] Guest G, MacQueen KM, Namey EE (2012). Applied Thematic Analysis.

[13] Berry NS (2014). Did we do good? NGOs, conflicts of interest and the evaluation of short-term medical missions in Solola, Guatemala. Soc Sci Med.

[14] Solheim J, Edwards P (2007). Planning a successful mission trip: the ins and the outs. J Emerg Nurs.

[15] Malterud K (2001). Qualitative research: standards, challenges and guidelines. Lancet..

